# Selective resource allocation may promote a sex ratio in pollinator fig wasps more beneficial for the host tree

**DOI:** 10.1038/srep35159

**Published:** 2016-10-12

**Authors:** Zhao-Tian Li, Yan-Qiong Peng, Xiao-Lan Wen, K. Charlotte Jandér

**Affiliations:** 1 State Key Laboratory of Genetic Resources and Evolution, Kunming Institute of Zoology, Chinese Academy of Sciences, Kunming 650223, Yunnan, China; 2 Kunming College of Life Science, University of Chinese Academy of Sciences, Kunming 650204, Yunnan, China; 3 Key Laboratory of Tropical Forest Ecology, Xishuangbanna Tropical Botanical Garden, Chinese Academy of Sciences, Menglun 666303, Yunnan, China; 4 Department of Organismic and Evolutionary Biology, 26 Oxford Street, Harvard University, Cambridge MA 02138, USA

## Abstract

Mutualisms play a key role in most ecosystems, yet the mechanisms that prevent overexploitation of the mutualistic relationship are still poorly understood. In the mutualism between fig trees and their pollinating wasps both partners depend on each other. Fig trees benefit from female wasps that disperse their pollen, whereas wasps frequently benefit from a higher ratio of male offspring. Here we use manipulative field experiments to address whether host trees (*Ficus racemosa*) can influence the offspring sex ratio of the pollinator wasp. We controlled wasp matings; virgin wasps can lay only male eggs. We found that virgin foundress wasps had fewer offspring than mated foundresses. This was not caused by virgin wasps having a shorter lifespan, or laying fewer eggs. Instead, male wasp larvae were more likely to die during development. Additionally, male eggs were deposited in flowers of equal style length to those of female eggs, yet emerged from galls with shorter pedicels than those of female wasps. We suggest that male larvae are either allocated less resources by the tree, or are less able to attract resources, during development. If the tree orchestrates this difference it would promote a more female-biased wasp brood, thus increasing the tree’s fitness.

Mutualisms are widespread and often have key ecosystem functions. Examples include pollinators that help flowering plants set fruit, symbiotic algae that help build coral reefs, nitrogen-fixing rhizobia in the root nodules of legumes, and gut bacteria that aid animals in nutrient uptake^1^,^2^,^3^. In a mutualism both partners benefit from the interaction. However, in the absence of fitness-aligning mechanisms between the partners such as vertical transmission of symbionts, or repeated interactions with automatic fitness benefits, other mechanisms are needed to prevent one partner from taking the fitness benefits without paying the costs^1^,^4^,^5^,^6^. In many mutualisms the host can selectively allocate more resources to those symbionts that provide the most benefits. For example, legumes have been shown to selectively allocate more resources to nodules containing rhizobia that are better at fixing nitrogen, and mycorrhizal fungi allocate more phosphorous to plants that provide them more carbon^7^,^8^,^9^,^10^.

A well-known and important model system for studying mutualism stability is that between fig trees (*Ficus* spp.) and their pollinating wasps (Hymenoptera: Aganoidae)^11^,^12^,^13^,^14^,^15^. Female fig wasps (foundresses) pollinate and oviposit in the flowers within the distinctive and enclosed inflorescences (formally syconia; hereafter figs) that define the genus *Ficus*. In monoecious fig species both seeds and wasps are produced in each fig, often hundreds of each. Each fig wasp larva develops within a single galled flower, thereby destroying a flower that otherwise could have been used for a seed. When female wasps emerge from their galls, they collect pollen from their natal fig^13^,^16^, then leave and disperse to a different, flowering tree where they can lay their eggs and pollinate^17^,^18^. The fig tree – fig wasp mutualism is therefore obligate because neither partner can reproduce without the other^19^.

There are several potential conflicts between the partners inherent in the fig tree – fig wasp mutualism. First, why do wasps spend time and energy pollinating their host? This conflict is not treated here, but seems to be solved by trees directing more resources to better pollinated figs, thereby increasing the fitness of foundresses that pollinate^13^,^14^,^20^,^21^,^22^,^23^. Second, why do not wasps lay eggs in all the flowers? This conflict is not treated here, and it is still not fully understood how it is resolved, although the outcome universally seems to favour the tree^13^. The third conflict is over what proportion of wasp-allocated flowers should be used to produce female vs. male wasp offspring. The tree pays a cost for producing wasps (both by the lost opportunity to make more seeds, and by the resources expended to raise each wasp), but benefits only from female wasps that disperse its pollen, and a minimum number of male wasps to dig an exit tunnel for the females. Wasps on the other hand try to optimize the sex ratio among their offspring according to the number of foundresses sharing the same fig, known as local mate competition^24^. When there is only one foundress she benefits from investing in only sufficient sons to impregnate the daughters and create an exit tunnel. As the number of foundresses increase, sons become more valuable and the optimal sex ratio approaches 50:50^24^,^25^,^26^,^27^. This increase in the production of male wasp offspring is not beneficial for the tree as each male is produced at the expense of a female. The sex ratio shift in fig wasps has been documented beautifully with theory and field data^25^,^26^,^27^,^28^, but how the resulting conflict between the tree and the wasps might be resolved has hitherto received little attention.

In this paper we start to address how the conflict over what proportion of wasp-allocated flowers is used for female wasps is resolved. Fig wasps are haplodiploid: fertilized eggs develop into diploid females and unfertilized eggs develop into haploid males^29^,^30^,^31^. By choosing to fertilize each egg (or not), the foundress can decide the sex of her offspring. The foundress therefore directly determines whether a flower is used for a male or female offspring when she lays her eggs. However, it is also possible that the tree might invest differently in male and female wasp galls; females are valuable for the tree as pollen dispersers, whereas males (other than the few needed to chew an exit tunnel for the females) are of no use for the tree. If male wasp larvae receive fewer resources from the tree during development than female larvae, or are otherwise selected against by the tree, this would lead to male larvae having a higher mortality rate than female larvae, thus creating a more female-biased brood than the foundress intended. Here we experimentally manipulate the sex ratio of wasp offspring by controlling the foundress’ mating behaviour – virgin foundresses can only lay male (unfertilized) eggs. We then investigate whether male wasp offspring have higher mortality during development than do female offspring. One potential explanation for higher mortality in male wasp larvae could be that male eggs are deposited in less favourable flowers than female eggs^32^. We therefore also assess whether male eggs are deposited in flowers further from the fig lumen than are female eggs. Such outer flowers have been suggested to have less space during development, be more exposed to parasites, and be less favourable to wasp development^32^,^33^,^34^,^35^,^36^,^37^. We further test whether our virgin treatment affects wasp lifespan and therefore the number of eggs a wasp may lay. We find that male offspring have a higher mortality than female offspring. A possible explanation for this is that the tree directs more resources to the more profitable female larvae, thereby promoting a more female-biased sex ratio in the wasp brood.

## Results

### Field experiments: reproductive output of mated vs. virgin foundresses

Treatment succeeded for 18 figs with mated foundresses (produced male and female offspring) and 11 figs with virgin foundresses (produced only male offspring). The remaining figs aborted during development, or treatment failed due to additional “wild” foundresses having entered, or what we thought were “virgin” foundresses were actually mated but we had failed to notice the tiny mating hole in the gall; such failed treatment figs were excluded from further analyses.

**Figure 1:**
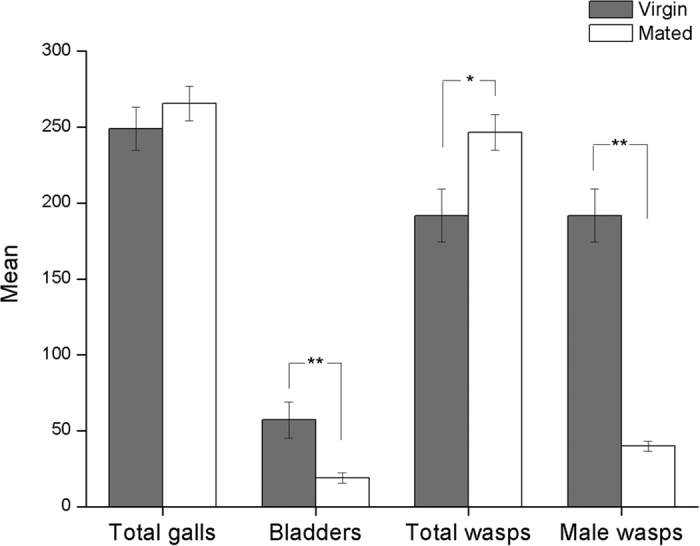
The mean number of total galls, bladders, total matured wasp offspring and male wasp offspring of mated (open bars) and virgin (filled bars) foundresses. Error bars represent 1 s.e.m. Mated and virgin foundresses produced almost the same number of galls (P = 0.374). However, offspring mortality during development (represented by bladders) was higher for virgin foundresses, resulting in fewer matured offspring overall. All offspring of virgin foundresses were male. *P < 0.05; **P < 0.01.

Mated and virgin foundresses initiated similar number of galls (mean ± s.e.m.: mated mean 265.50 ± 11.43; virgin mean 248.91 ± 14.22; t_27_ = −0.90, p = 0.374; Fig. 1), indicating that they laid similar number of eggs (assuming that each gall originated from the deposition of an egg and associated fluids, which is consistent with all available evidence^36^,^38^,^39^,^40^). However, the offspring of virgin foundresses were significantly more likely to fail to develop, creating a higher number of empty bladders when foundresses were virgin (mean 57.18 ± 11.86) than when mated (mean 19.00 ± 3.39) (Mann-Whitney U-test, test statistic 26.5, P = 0.001; Fig. 1). Therefore, significantly fewer wasp offspring emerged as adults from figs with virgin foundresses (mean 191.73 ± 7.40) than from figs with mated foundresses (mean 246.50 ± 11.77) (Mann-Whitney U-test, test statistic 149, P = 0.024; Fig. 1). The proportion of galls that developed to maturity to release adult wasps was 77% for virgin foundresses but 93% for mated foundresses. As expected, there were significantly more male offspring emerging from figs with virgin foundresses (mean 191.73 ± 17.40) than from figs with mated foundresses (39.83 ± 3.21) (Mann-Whitney U-test, test statistic 0.0, P < 0.001; Fig. 1).

We also compared the number of seeds in each treatment. There was no significant difference between the two groups: figs with virgin foundresses contained on average 764.9 ± 129.8 seeds, and figs with mated foundresses contained 544.2 ± 84.9 seeds (t_27_ = 1.49, P = 0.15). Therefore the reduction in offspring numbers of virgin foundresses that we find here cannot be explained by lack of pollination^20^,^41^. Despite containing no fewer seeds, figs with virgin foundresses tended to be smaller at maturity than figs with mated foundresses. Figs with virgin foundresses had a diameter at maturity of 3.36 ± 0.14 cm, whereas figs with mated foundresses had a diameter of 3.66 ± 0.14 cm (t-test, t_12.65_ = −1.96, P = 0.072).

### Male galls develop shorter pedicels than female galls

**Figure 2:**
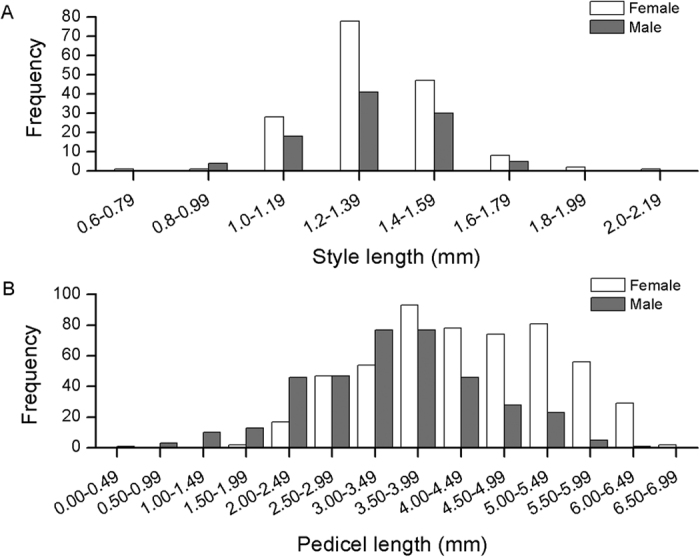
The (**A**) style length, and (**B**) pedicel length of galls containing male (filled bars) or female (open bars) wasps. (**A**) Galls containing male wasps did not have longer styles than galls containing female wasps, suggesting that unfertilized (male) and fertilized (female) eggs were oviposited into flowers with similar style lengths. (**B**) At maturity, galls containing male wasps had shorter pedicels than galls containing female wasps.

**Figure 3:**
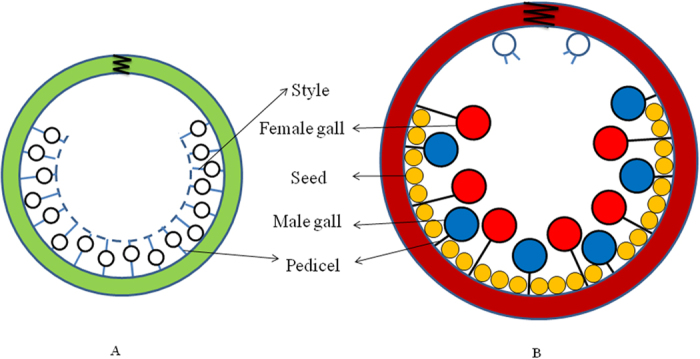
Diagram of figs at two different stages showing how flowers are packed inside a fig. (**A**) Female flower phase: Flowers with short styles have long pedicels, and vice versa; (**B**) Male flower phase: Styles have withered. Galls containing female wasps tend to have relatively longer pedicels, galls containing male wasps tend to have relatively shorter pedicels. Seeds have very short pedicels. Differences in pedicel lengths have here been exaggerated for illustrational purposes. Our measurements of style and pedicel lengths were done at intermediate maturation stages (see text).

Foundresses seemed to deposit male and female eggs in flowers of equal style length: male mean 1.31 ± 0.02 mm, n = 98; female mean 1.34 ± 0.01 mm, n = 166). The GLM revealed that the length of styles did not depend on the sex of the wasp inside each gall (F_1, 257_ = 1.05, P = 0.31; Fig. 2), but depended on the individual fig in which they were present (F_5, 257_ = 8.96, P < 0.001). However, later in development when wasps were close to emerging, male galls had shorter pedicel (mean ± SE:3.41 ± 0.05 mm) than galls containing female wasps (mean ± SE:4.34 ± 0.05 mm). The GLM revealed that the length of the pedicel was strongly influenced by the sex of the wasp inside its gall (F_1, 892_ = 187.72, P < 0.001; Fig. 2), and also of the individual syconia (F_14, 892_ = 3.76, P < 0.001) and tree (F_3, 14.02_ = 4.80, P < 0.001). This means that at the end of fig development, male galls were on average located closer to the fig wall, and female galls were closer to the lumen (Fig. 3). Because style length did not initially differ, and style length and pedicel length are strongly correlated in early fig development^32^, this data suggest that the difference in pedicel length between male and female galls develops during fig development.

### The effect of mating and humidity on lifespan

**Figure 4:**
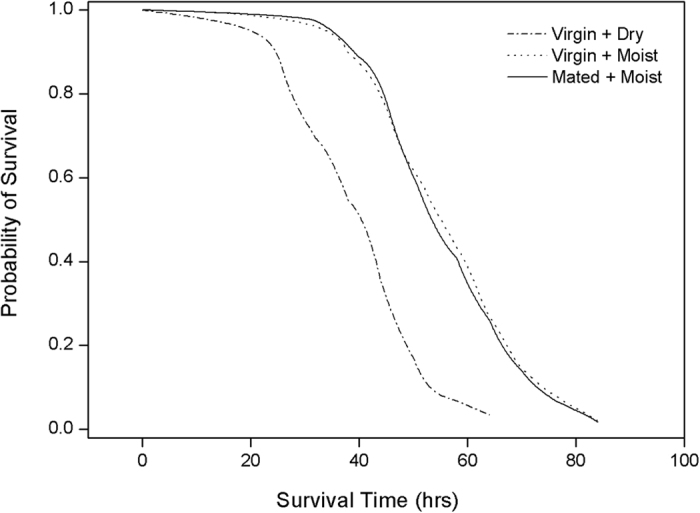
The survival curves of mated and virgin wasps at different humidity levels. Mated wasps (solid line) did not live longer than virgin wasps (dotted line) in a moist environment. Wasps survived longer in a moist than in a dry environment.

Mating status did not affect the lifespan of female wasps in a moist environment: virgin wasps lived just as long (56.9 ± 1.3 h) as mated wasps (56.4 ± 1.2 h) (paired t-test, t_4_ = 0.42, P = 0.70; Kaplan-Meier, Breslow test χ^2^_1_ = 0.11, P = 0.74; Fig. 4). Confirming previous findings^35^, virgin wasps in a dry environment lived much shorter (40.7 ± 1.3 h) than virgin wasps in a moist environment (56.9 ± 1.3 h) (paired t-test, t_4_ = −10.61, P <  0.001; Fig. 4).

## Discussion

In summary, we have documented that: 1) Virgin foundress fig wasps have fewer offspring than mated foundresses. 2) Male fig wasp larvae are more likely to die during development. 3) Male eggs are deposited in flowers of equal style length to those of female eggs, yet emerge from galls with shorter pedicels than those of female offspring. These findings lead us to suggest that male larvae are either allocated less resources by the tree, or are less able to attract resources from the tree during development. Such differential access to resources would result in a more female-biased sex ratio in the wasp brood, thus increasing the tree’s fitness.

We found that virgin foundresses had fewer total offspring that developed to adults than did mated foundresses. Virgin foundresses did not have shorter lifespan or deposited fewer eggs than mated foundresses. Instead, a larger proportion of galls initiated by virgin foundresses failed to develop and thus turned into bladders (galls that are empty, or contain a dead, partly developed wasp larva). Three alternate hypotheses might explain this higher mortality in male than in female larvae: H_1_: There is higher innate mortality in male than in female larvae. H_2_: The host tree directs fewer resources to developing male larvae. H_3_: Developing male larvae are less able to attract resources from the tree. We discuss these three hypotheses below.

The simplest hypothesis is that there is higher innate mortality in male than in female larvae. Could male haploidy increase their innate mortality rate? However, where it has been measured in other haplodiploid Hymenoptera, the mortality of male and female brood is similar: Honey bee drones have a mortality from egg to adult of 44% vs queens 47%^42^,^43^. The polyembryonic wasp *Copidosoma floridanum* lays broods that are either all male, all female, or mixed. Broods that were all male were as large as broods consisting of all females^44^. We therefore do not find it compelling that male fig wasp larvae would have innate higher mortality than female larvae. In fact, a recent study on *F. racemosa* argues that the haploid male fig wasp larvae are more resilient against starvation than are female wasp larvae^41^.

The remaining two hypotheses (H_2_: The host tree directs fewer resources to developing male larvae, and H_3_: Developing male larvae are less able to attract resources from the tree) both involve males accessing less resource than female larvae. We think these two hypotheses are likely explanations for two reasons: 1) Selective resource allocation to more profitable tissues is prevalent in plants, and is already documented in the fig tree – fig wasp mutualism^23^. 2) We found male eggs to be deposited in flowers of equal style length to those of female eggs, yet males emerge from galls with shorter pedicels than those of female offspring. This suggests that galls containing female larvae grow longer pedicels during their development. The previously documented fact that larger wasps emerge from galls with longer pedicels^15^,^32^,^34^ may not be solely because they were deposited in flowers with short styles (long pedicels) – instead both large wasp size and growth of a long pedicel may be responses to certain wasp larvae being able to attract more resources from (or be allocated more resources by) their host than others. Detailed studies examining the chemical conversation between the developing wasps and the tree are needed to disentangle whether the difference in resource availability is caused by the tree or the developing wasps.

Female wasp offspring are more beneficial for the host tree than male wasp offspring, because female wasps disperse the pollen from their natal fig and therefore form the tree’s male fitness component. It would therefore be beneficial for the tree to be able to selectively allocate more resources to female than to male fig wasp larvae during their development. Such selective resource allocation could lead to: 1) higher mortality among developing male larvae (as documented here), 2) smaller size of male adult wasps than female adult wasps (this is true in *F. racemosa* (Li Z-T, unpublished data)), 3) shorter pedicel length for male galls (as documented here), 4) smaller mature fig fruit when it contains only male wasps (as documented here). Such findings would be completely analogous to the effects of reduced resource allocation we see to unpollinated figs compared to pollinated figs, where unpollinated figs are smaller at maturity than pollinated figs, and wasp larvae in unpollinated figs have a higher mortality and/or develop into smaller adults^22^,^23^. While such selective resource allocation is not expected to select for any changes in the sex ratio of the deposited eggs (differential mortality of offspring after parental investment has ended is not expected to change primary sex ratios^45^), it would nevertheless reduce the amount of resources that the tree invests in less profitable male wasps, thus increasing the tree’s fitness.

The selective resource allocation proposed here would be most effective if it acted on the level of individual flowers, distinguishing male galls from female galls in figs that contain both^21^. If it instead acted on the level of the entire fig fruit it would save the tree some resources when a fig was oviposited by only virgin foundresses, but would not be as effective when figs have high male sex ratios among the wasp offspring due to multiple foundresses (where both male and female larvae are present). Although we do not have the data to distinguish the two here, it would be straightforward to test in future experiments: If resource allocation is on the fig level, male wasp larvae in figs with mated foundresses could free-ride on the resources allocated to/pulled in by female wasp larvae, and therefore would be larger than male wasps that develop in figs with only virgin foundresses. If resource allocation instead acts on the flower level, male wasps in figs with mated foundresses would be just as small as male wasps in figs with virgin foundresses.

In the fig tree – fig wasp mutualism, it seems that the trees are dominating in the first two conflicts (most wasps do pollinate, and wasps do not lay eggs in all flowers)^13^,^14^,^46^,^47^,^48^. In contrast, the wasp offspring sex ratio is much closer to what is optimal for the wasps than the trees. Nevertheless, wasp sex ratios are generally more female biased than predicted by optimality models^26^,^28^. Because it is clear that the tree would benefit from a more female biased sex ratio among the wasps developing in its figs, it does not seem implausible that trees have been selected to distinguish between male and female larvae and allocate resources accordingly, thus manipulating the sex ratios of the wasp broods it rears.

## Methods

### Study system and natural history

*Ceratosolen fusciceps* is the pollinator of *Ficus racemosa* in the prefecture of Xishuangbanna in southwestern China^49^,^50^. *F. racemosa* is a monoecious species that has seeds and pollen developing in the same fig; wasps are active pollinators^12^,^51^. The work was carried out at Menglun town and Xishuangbanna Tropical Botanical Garden (XTBG). All wasp introduction experiments were carried out on three *F. racemosa* fig trees; foundresses for the mated and virgin treatments were collected from nearly mature figs on different trees. Collections for style and pedicel length were made on four additional trees.

In receptive figs, flowers are packed in multiple layers with stigmas reaching to the inner cavity (Fig. 3). Foundress fig wasps lay one egg per flower through the style into the ovary^40^. The flower is attached to the fig wall through a pedicel; style and pedicel length are inversely correlated early in fig development, and pedicels elongate during fig development^32^,^52^,^53^ (Fig. 3). The style lengths of flowers in the syconia of monoecious *Ficus* are highly variable^11^,^54^ (Fig. 3). Most long styled flowers remain unexploited by pollinator wasps and develop into seeds, whereas the short-styled inner flowers are often used for pollinator wasp development^34^,^55^,^56^,^57^. Inner flowers seem to be more favourable for wasp development, producing larger wasps and wasps that emerge sooner^15^,^32^,^34^. In previously examined fig species, male wasp eggs are predominantly deposited first, often in short-styled flowers^57^,^58^,^59^,^60^. When the fig is mature, male wasp offspring emerge first. Males move around inside the fig, bite small holes in the galls that contain female wasps and insert their elongated abdomen to mate with each female while she is still in her gall, then move to the next female gall. Each mated female enlarges the hole and emerges from her gall to collect pollen. When all females are mated the males chew a tunnel through the fig wall so that females can exit. Males are wingless and end their life in their natal fig; females collect pollen, then disperse to a different tree with receptive figs, where they pollinate and lay their eggs before they die.

### Experimental introductions of mated and virgin fig wasps

We prevented uncontrolled pollination of pre-receptive experimental figs by enclosing branches in fine mesh bags^61^. To prepare mated and virgin wasps we collected figs close to maturity, opened them, and chose figs in which male wasps had emerged but none or few female wasps had yet emerged. From these figs, we, using a dissecting microscope and fine forceps, removed 1) galls with a hole (mated) but where females had not yet emerged, and 2) galls with no visible hole (virgin). We chose figs where males had just started mating with the females, so that we were able to collect both types of galls (mated and unmated) from the inner layer of flowers within the same fig, positioned closely to each other. The extracted galls with mated or virgin female wasps were put in separate vials with 1 cm wet cotton at the bottom to retain moisture^35^. To facilitate the emergence of virgin wasps we made a small hole in their galls using fine forceps. The tubes were stored at room temperature and wasps were allowed to emerge from their galls naturally. When experimental figs became receptive, each fig was randomly assigned to one of two treatments: (1) one mated wasp and one natural ovipositor-excised wasp, or (2) one virgin wasp and one natural ovipositor-excised wasp, and wasps were introduced using standard methods^20^. The ovipositor-excised wasp was introduced to provide pollen in order to avoid the fig abortion that commonly occurs in *F. racemosa* when there is not sufficient pollination^41^. The ovipositor- excised wasps originated from wild wasps collected around the flowering tree, their ovipositors were cut using a scalpel and they were immediately introduced into the experimental figs (ca 6 hours after the mated/unmated wasps). To minimize variability we chose wasps that were as close in size as possible for the introduction experiments^62^,^63^. We introduced 30 mated foundresses and 50 virgin foundresses. We then replaced the bags around the figs and allowed the wasp offspring to develop on the tree until maturity (approximately 33 days). When figs were mature, we collected the figs and brought them to the lab to measure their diameter to the nearest 0.01 mm using a caliper, and dissected them to count the number and sex of wasp offspring, bladders (empty galls due to wasp larvae failing to develop), and seeds^23^,^40^,^49^. We assumed that each gall originated from the deposition of an egg and associated fluids, which is consistent with all available evidence^36^,^38^,^39^,^40^.

### The length of style and pedicel of male and female galls

To measure the length of the styles and pedicels of male and female galls we collected naturally pollinated figs at two different developmental stages (Fig. 3).

For style length measurements we collected figs at an early stage of development because the style is easier to measure early in fig development^32^, but at a stage when wasp larvae were sufficiently developed so that males could be distinguished from females, ca 20 days from pollination. We collected 6 figs and for each fig cut a longitudinal slice from one pole to another. Using a dissecting scope and fine forceps we haphazardly removed individual galls from this slice. For each gall, we measured the style length to the nearest 2.5 μm using a reticle mounted on the microscope, then opened the gall to record the sex of the wasp within. In total, we measured styles on 98 male galls and 166 female galls (Table 1). To measure pedicel length we waited until figs were close to maturation, ca 31 days from pollination. As with the style length measurements, we haphazardly removed galls along a longitudinal slice of the fig and measured their pedicel length (Fig. 3), then opened each gall to determine the sex of the wasp within. In total, we measured pedicels on 377 male galls and 533 female galls from 18 figs originating from four different trees (Table 2).

### Lifespan of mated and virgin wasps

In insects, seminal fluids are known to be able to either prolong or shorten a female’s lifespan^64^,^65^,^66^. If seminal fluids affected the lifespan of female fig wasps it would confound our experiment. To find out whether seminal fluids could prolong or shorten a female fig wasp’s lifespan we collected mated and virgin female wasps from five figs as described earlier. We haphazardly split the virgin wasps from each fig into two vials, and randomly allocated them to either a moist (1 cm wet cotton wool in vial; n = 88)) or a dry environment (dry cotton wool in vial; n = 97), as humidity can affect the lifespan of fig wasps^35^. Mated wasps were only exposed to the moist treatment (n = 114). Each fig contributed 11 to 32 wasps to each treatment; wasps from different figs were kept in separate vials. We stored all vials at room temperature and recorded the number of dead individuals in each vial every two hours until the last wasp was dead.

### Statistical methods

In the field experiments with introduced foundresses, we compared the number of offspring of each sex, bladders, and seeds from figs with mated versus virgin foundresses using either t-tests (when normally distributed) or Mann-Whitney U tests.

We analysed style length with a GLM with style length as the dependent variable, sex of the wasp (male or female) as a fixed factor, and fig as a random factor. We analysed pedicel length with a GLM with pedicel length as the dependent variable, sex of the wasp (male or female) as a fixed factor, and tree, and fig nested within tree, as random factors.

When comparing the lifespan of virgin wasps in a dry environment with virgin wasps in a moist environment we used a paired t-test with the mean lifespan of wasps originating from each fig (n = 5) as an independent sample. This test ensures independence among samples and is conservative. In contrast, when comparing the mean lifespan of virgin wasps in a moist environment with that of mated wasps in a moist environment we wanted as high power as possible to avoid type II errors. For that reason we used the higher powered Kaplan-Meier test in addition to the conservative low-powered paired t-test. In the Kaplan-Meier test each wasp is treated as an independent data point, and sample sizes are therefore much higher. Treating wasps emerging from the same fig as independent data points is not entirely correct because they shared developmental environment, and additionally may be sisters. However, analysing our data this way increases power and therefore minimizes type II errors; if we fail to find a difference between the groups using the high powered Kaplan-Meier test we can be confident that there is no difference.

All statistical tests were performed in SPSS 19 and were two-tailed.

## Additional Information

**How to cite this article**: Li, Z.-T. *et al*. Selective resource allocation may promote a sex ratio in pollinator fig wasps more beneficial for the host tree. *Sci. Rep.* **6**, 35159; doi: 10.1038/srep35159 (2016).

## Figures and Tables

**Table 1:** Sample sizes for style length measurements in naturally pollinated figs of *F. racemosa*.

Fig ID	Male galls (n)	Female galls (n)
1	31	40
2	3	11
3	5	20
4	3	15
5	22	45
6	34	35

**Table 2:** Sample sizes for pedicel length measurements in naturally pollinated figs from four different trees of *F. racemosa*.

Tree ID	Fig ID	Male galls (n)	Female galls (n)
1	1	20	25
	2	22	28
	3	23	25
	4	21	31
	5	20	32
2	1	20	29
	2	24	25
	3	21	36
3	1	21	35
	2	13	27
	3	22	32
	4	22	35
	5	21	38
4	1	22	29
	2	21	25
	3	20	30
	4	21	30
	5	22	22
